# hnRNP A1 controls *MIR17HG* alternative splicing and maturation of *miR-19a*

**DOI:** 10.1007/s11033-026-12580-6

**Published:** 2026-08-12

**Authors:** Vivian Maltese Munhoz, Letícia Oliveira Rojas Cruz, Patricia Pereira Coltri

**Affiliations:** Departamento de Biologia Celular e do Desenvolvimento, Instituto de Ciências Biomédicas, Universidade de São Paulo, São Paulo, 05508-000 SP Brazil

**Keywords:** RBP, MIR17HG, *miR-19a*, *miR-17-92*, Alternative splicing

## Abstract

**Background:**

Alternative splicing is a key process in the regulation of gene expression in eukaryotes. During transcription, the spliceosome machinery assembles onto precursor messenger RNA (pre-mRNA) conserved regions, promoting intron removal and exon ligation. This process is guided and controlled by the association of RNA-binding proteins (RBPs) in the pre-mRNAs. These proteins mediate splice-site choice controlling the splicing profile observed in different cells. The heterogeneous ribonucleoproteins (hnRNPs) A1 and K have previously been associated with intronic regions containing microRNAs (miRNAs). We previously showed hnRNP A1 overexpression increases invasion and cell growth in thyroid carcinoma cells through association with *miR-17a* and *miR-18a*. *MIR17HG* is the host gene for the *miR-17-92* miRNA cluster, which is encoded within one of its introns. In this work, we investigated the participation of hnRNP A1 and hnRNP K in *MIR17HG* alternative splicing.

**Methods and Results:**

Overexpression of hnRNP A1 and hnRNP K was performed using pFLAG system in HEK-293FT cells. Reverse transcription quantitative PCR (RT-qPCR) analysis indicated higher levels of MIR17HG-i3 isoforms, which correlated with increase on *pre-miR-19a* generation upon hnRNP A1 overexpression. Surprisingly, the same pattern was not observed in cells overexpressing hnRNP K. Higher concentration of functional *miR-19a* in hnRNP A1 overexpressing cells was confirmed by luciferase reporter assays.

**Conclusions:**

These results indicate that hnRNP A1 modulates *MIR17HG* splicing, with downstream effects on *miR-19a* biogenesis and maturation.

**Supplementary Information:**

The online version contains supplementary material available at 10.1007/s11033-026-12580-6.

## Introduction

Pre-mRNA splicing is a co-transcriptional process responsible for removing introns and joining exons into mature messenger RNA (mRNA) sequences in eukaryotes. The reaction depends on recognition of splice sites, conserved sequences located on the borders of introns and exons, followed by intron removal and exon ligation [1]. A specialized 2 MDa macromolecular machinery named spliceosome catalyzes the splicing reaction through assembling small nuclear RNAs (snRNAs) and proteins within the transcribed introns [1, 2]. The spliceosome is assembled upon every transcribed intron guided by regulatory proteins and RNAs. During this process, splice site choice is important for exon and intron definition, as well as generation of alternative mRNAs. In addition to spliceosome core components, RNA-binding proteins guide spliceosome assembly to splice sites, facilitating or blocking generation of specific alternative transcripts through alternative splicing [3].

Alternative splicing is essential for the regulation of human gene expression. The use of different splice sites along the pre-mRNAs promotes inclusion of introns or exon skipping resulting in mRNAs with variable exon-intron combinations. These transcripts define specific cellular phenotypes and increase protein diversity. This process is essential for cellular specialization, allowing different cells to maintain a splicing profile capable of inducing specific phenotypes. Additionally, alternative splicing can lead to disruption of exon reading frames, generating truncated or dysfunctional proteins, as well as unstable mRNAs which are rapidly degraded upon nuclear mechanisms of surveillance [4–6]. The selection and choice of alternative splice sites is mostly mediated by RBPs, resulting in diverse mature mRNA sequences. The abundance of specific mRNA isoforms, therefore, is related to the concentration and activity of these regulatory proteins. The family of hnRNPs is well known for playing essential roles in alternative splicing regulation [7–10]. hnRNP A1 is composed of two conserved RRMs (RNA recognition motifs), important for mediating RNA binding, and a RGG box (glycine-rich region), which possibly facilitates its interactions with other hnRNPs [7, 11, 12]. hnRNP A1 shows a pro-tumoral phenotype in different cell types, promoting cell growth, migration and invasion [13–15]. We previously reported hnRNP A1 associates with *miR-17a* and *miR-18a* in papillary thyroid cancer cells leading to increased cell growth and cell invasion [15, 16]. This RBP is also involved with processing of important oncogenic miRNAs, such as *miR-24* and *let-7a*, directly associating its activity with tumor development [17]. Previous work using *in vivo* cross-linking and immunoprecipitation (CLIP) analysis in HeLa cells reported that hnRNP A1 binds to *pri-miR-18a*, acting as an auxiliary factor for miRNA processing [18]. hnRNP K is composed of three KH domains, important for mediating association with poly-C RNA sequences [7]. This protein shows both oncogenic and suppressive phenotype in different cell types, affecting cell growth, migration and apoptosis [19, 20]. It is widely recognized as a tumor suppressor in acute leukemia [19]. However, it is also known as oncogenic in colorectal and gastric cancers [20–22]. ENCODE data with CLIP analysis performed in K562 and HepG2 cell lines indicated that both hnRNP A1 and hnRNP K bind to *MIR17HG*, suggesting these RBPs might be associated with alternative splicing of this gene [23, 24].

More than 70% of human miRNAs are encoded within introns [25]. The oncogenic miRNA cluster *miR-17-92* is in intron 3 of *MIR17HG* and encodes six independent miRNAs already associated with tumorigenesis in different cell types [26]. *MIR17HG* is a non-coding transcript that undergoes alternative splicing, generating multiple isoforms with distinct exon and/or intron compositions. Among these, specific isoforms retain intron 3, which contain the *miR-17-92* cluster. In this study, isoforms retaining intron 3 will be referred to as MIR17HG-i3, whereas those lacking intron 3 will be designated MIR17HG-Δi3. We also analyzed the MIR17HG-standard expression, which comprises different isoforms of *MIR17HG*. Biogenesis and maturation of *miR-17-92* miRNAs varies across different cell types, and the individual miRNAs are independently processed, leading to either pro- or anti-tumoral characteristics in different cells [27, 28]. Among them, *miR-19a* is considered an oncogenic miRNA in different cancers, such as colorectal, thyroid and gastric cancers, osteosarcoma and lymphoma [27, 29–31]. In these cells, *miR-19a* has multiple targets, including PTEN [27, 32] and Wnt1, both involved in cell cycle regulation [33], and RAP1B, a Ras-related GTPase protein related to cell adhesion and growth, which has been associated with poor prognosis of different tumors [34, 35]. An extensive miRNA network controls RAP1B in different cells, including *miR-19a* in papillary thyroid cells [29].            

Previous work from our group revealed that hnRNP A1 stimulates cell growth, migration and invasion in papillary thyroid tumor cells [15]. hnRNP A1 overexpression immunoprecipitated *miR-17a* and *miR-18a* and enhanced *miR-19a* expression in these cells, all of which belong to the *miR-17-92* cluster and are directly related to oncogenesis [16, 36, 37]. Given the established role of hnRNP proteins in alternative splicing regulation, we hypothesized that hnRNP A1 and hnRNP K could influence the splicing pattern of *MIR17HG*, leading to changes in the abundance of isoforms containing the *miR-17-92* cluster. Consequently, alterations in MIR17HG-i3 expression could affect the biogenesis of the encoded miRNAs, potentially affecting cellular phenotypes relevant to oncogenesis. Consistent with this hypothesis, our results indicated that both hnRNP A1 and K modulate *MIR17HG* alternative splicing pattern. Overexpression of these RBPs increased the generation of MIR17HG-i3 isoforms, which contain the miRNA cluster. Moreover, hnRNP A1 overexpression was associated with increased *pre-miR-19a* expression, and generation of functional *miR-19a* molecules, as demonstrated by luciferase reporter assays. While hnRNP K exerted a similar effect on MIR17HG-i3 abundance, no corresponding increase in *pre-miR-19a* expression was observed.

### Methods

### Cell culture and transfection

HEK-293FT cells (RRID: CVCL_6911), derived from human embryonic kidney, were obtained from Invitrogen (catalog#R70007). The cell line was free of bacterial or mycoplasma contamination, and cellular morphology was assessed prior to the experiments. This cell line was selected due to expression of *miR-17-92* miRNAs. Appropriate controls using non-transfected cells or cells transfected with empty vectors were performed for all experiments. The cell line was cultivated in Dulbecco’s Modified Eagle’s Medium (DMEM) High Glucose (Thermo) supplemented with 10% fetal bovine serum (FBS; Thermo), 100 U/mL penicillin (GIBCO), 100 µg/mL streptomycin (GIBCO) and 0.25 µg/ mL amphotericin B (HyClone) in 100 mm Petri dishes and maintained at 37 °C in a humidified incubator with 5% CO_2_. To perform the luciferase assays, cells were cultivated in 24 well-plates (Corning). At approximately 80% confluence, cells were detached using 1X trypsin-EDTA solution (Thermo).

To overexpress the hnRNP A1 and hnRNP K proteins, the plasmids pFLAG-hnRNP A1 [15] and pFLAG-hnRNP K were transfected using Lipofectamine 2000 (Thermo) on 70–80% confluent cells, according to the manufacturer’s instructions. No selection was performed, and cells were collected 96 h after transfection. Empty pFLAG was transfected as control under the same conditions.

### RNA extraction and RT-qPCR

Total RNA was extracted from cell pellets collected 96 hours after transfection. Pellets were resuspended in Trizol reagent (Thermo), followed by extraction using chloroform, and further precipitation with 3 M sodium acetate pH 5.2 and ethanol. According to the manufacturer’s instructions, cDNAs were synthesized using SuperScript IV Reverse Transcriptase kit (Thermo) and random primers. Quantification to assess overexpression of hnRNPs, alternative splicing isoforms and pre-miRNA levels were performed by RT-qPCR using SYBR Green reagent (Thermo) and specific primers (Table 1). Relative gene expression was calculated by 2^−∆∆Ct^ method [38]. Fold changes in gene expression were normalized using *ACTB* or *RNU6B* genes, as indicated for each assay (Table 1).

**Table 1:** Primers used in this study

Primer	Forward Primer	Reverse Primer
*ACTB*	ACCTTCTACAATGAGCTGCG	CCTGGATAGCAACGTACATGG
*RNU6B*	CTCGCTTCGGCAGCACATATAC	GGAACGCTTCACGAATTTGCGTG
hnRNP A1	TGCAAAACCACGAAACCAAG	GCTTCCCTGTCACTTCTCTG
hnRNP K	GGACGTGCACAGCCTTATGA	CGCGACGGTCATCAAACATC
MIR17HG-standard	CGGTCGTAGTAAAGCGCAGG	CTGGTGCAGTTAGGTCCACG
MIR17HG-Δi3	TGGTATTTGCTAAGTGGAAGCC	CAGGTCTGCCTTTTAGAGGAAA
MIR17HG-i3	GGTACACATGGACTAAATTGCCT	GGAAGTGGTGGCTCTTCCAA
*pre-miR-19a*	ATAGCCTCGAGGCAGTCCTCTGTTAG	ATGATAAGCTTGCAGGCCACCATCAG

### Western blot

Overexpression of hnRNP A1 and hnRNP K was confirmed by western blot. Cells were collected 96 hours after transfection for protein extract preparation using RIPA buffer (50 mM Tris-HCl (pH 7.4), 150 mM NaCl, 1 mM EDTA, 1% NP-40, 0.25% sodium deoxycholate), supplemented with 0.5 mM DTT and 1X protease inhibitor cocktail (100X, AMRESCO) and Douncer homogenizer. Protein concentrations were determined using Bradford assay [39]. For protein separation, 20 µg of total protein from whole-cell extract samples were loaded onto a 10% SDS-polyacrylamide gel and subjected to electrophoresis. Separated proteins were transferred onto a polyvinylidene difluoride membrane, which was subsequently blocked with a 5% bovine serum albumin (BSA) in TBS-T 1X (TBS with 0.01% Tween-20). The membrane was incubated with primary antibodies diluted in TBS-T, anti-hnRNP A1 (Proteintech; 1:5,000 dilution), anti-hnRNP K (Proteintech; 1:5,000 dilution) and anti-β-actin (Sigma; 1:10,000 dilution). Following washes with TBS-T, the membrane was incubated with secondary antibodies anti-IgG rabbit (1:10,000 dilution) (Sigma), for hnRNP A1 and hnRNP K, and anti-IgG mouse (1:10,000 dilution) (Sigma) for β-actin. The chemiluminescent signal was detected using the Amersham™ Imager 600 Series, following a 3-minute exposure. Densitometric analysis of protein bands was conducted using ImageJ software (version 1.54 g; https://imagej.net/ij/). Protein expression levels were normalized using β-actin, which served as a loading control. Graphical representation of the data was generated using GraphPad Prism software (version 8.02; https://www.graphpad.com).

### Luciferase reporter assay

After transfection with pFLAG and pFLAG-hnRNP A1, HEK-293FT cells were cultivated until 80% confluence in a 24-well plate in five independent biological replicates. For the reporter assay, these cells were co-transfected with pmirGLO-RAP1B, pmirGLO-mismatch [40] or the empty pmirGLO, using Lipofectamine 2000 as described before.

To quantify and compare different luminescence intensities, we used the Dual-Luciferase Reporter Assay Kit (Promega). For each biological replicate, technical triplicates were performed in a 96-well black plate with clear bottom (Corning). Firefly luciferase (FL) was normalized by activity of the control Renilla luciferase (RL) (FL/RL), as recommended by the manufacturer (Promega). Since empty pmirGLO is not regulated by miRNAs, its FL/RL ratio was used as internal control for each group, providing the number of relative light units (RLU) for each experimental condition. Considering the importance of different cellular backgrounds to analyze the data, FL/RL ratios were normalized to the corresponding ratio of the empty reporter vector within each cell condition. Graphic representation of the RLU values were plotted using the GraphPad Prism software (version 8.02; https://www.graphpad.com).

### Cell proliferation

To address cell proliferation, HEK-293FT cells transfected with pFLAG-hnRNP A1, pFLAG-hnRNP K and empty pFLAG were seeded at 5 × 10^4^ cells/mL in 6-well plates (Corning). After plating, cells were cultured for 24, 48, 72, and 96 hours. Cells were collected by trypsinization using 1X trypsin-EDTA solution (Thermo) and resuspended in 1 mL of DMEM high glucose (GIBCO) supplemented with 10% fetal bovine serum (Thermo). Cell counting was performed upon staining with 0.4% Trypan blue (AMRESCO). Cell viability was assessed using the Countess 3 Automated Cell Counter (Invitrogen), with each sample analyzed in duplicate.

### Statistical analysis

RT-qPCR and cell proliferation assay results obtained from HEK-293FT cells overexpressing hnRNP A1 and hnRNP K were analyzed based on three and four independent biological replicates, respectively. Data normality was assessed using the Shapiro-Wilk test, followed by Student’s t-test. For the luciferase reporter assay, RLU from five independent biological replicates were analyzed using two-way ANOVA, followed by Turkey’s multiple comparisons post-test.

All data were analyzed using the GraphPad Prism software platform 8.02 version (https://www.graphpad.com). Each analysis was plotted as mean ± standard deviation (SD). Differences were considered statistically significant when p-values were lower than 0.05. In these graphs, a single asterisk (*) denotes *p* < 0.05, double asterisks (**) indicate *p* < 0.01, and triple asterisks (***) correspond to *p* < 0.001. The abbreviation ‘ns’ (not significant) indicates that the observed difference did not reach statistical significance.

## Results

### hnRNP A1 and hnRNP K modulate splicing pattern of *MIR17HG*

**Fig. 1:**
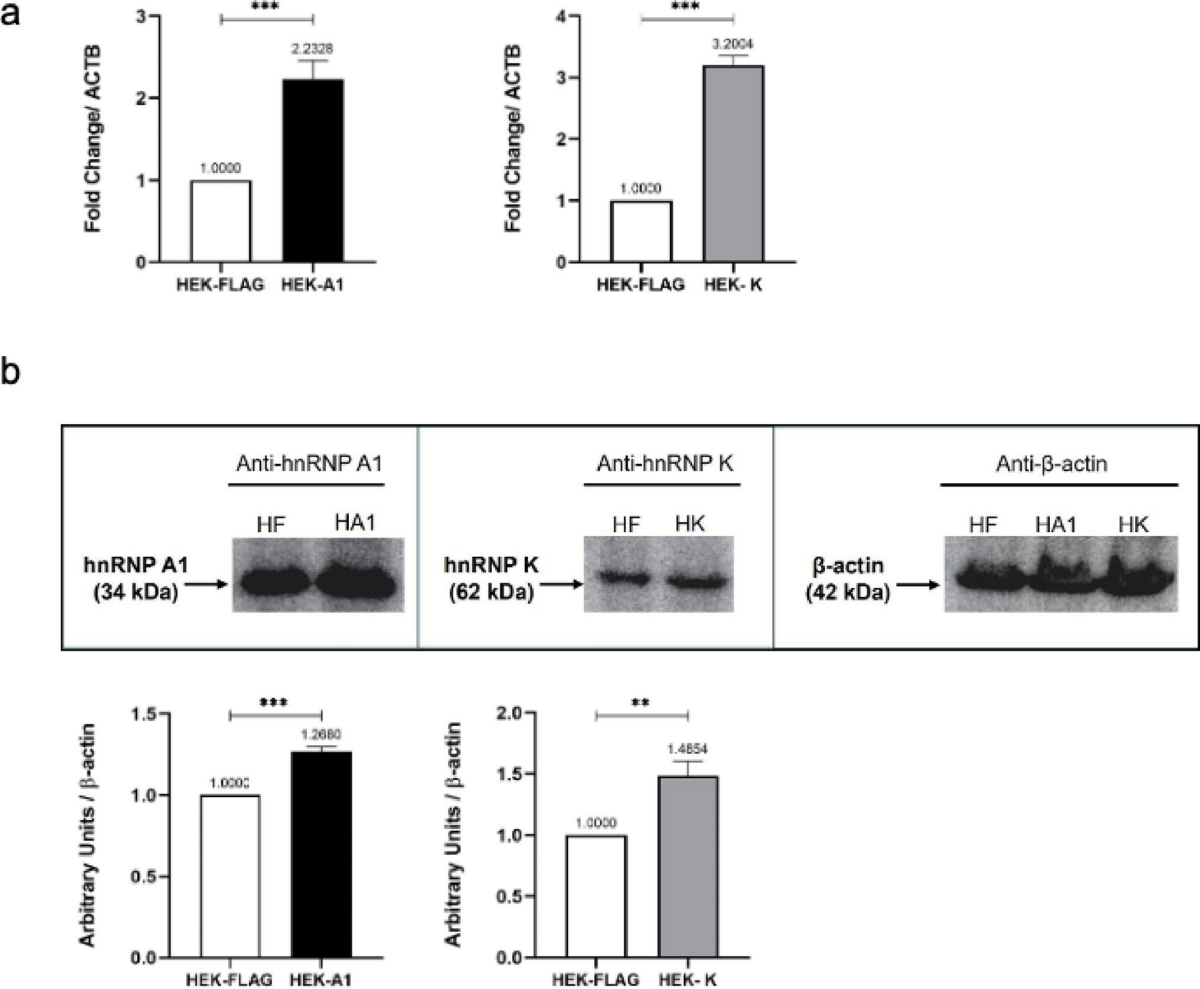
Confirmation of hnRNP A1 and hnRNP K overexpression in HEK-293FT cells. (**a**) RT-qPCR and (**b**) western blot were performed in HEK-293FT cells transfected with pFLAG-hnRNP A1 (HEK-A1/HA1; black bars) and pFLAG-hnRNP K (HEK-K/HK; grey bars) vectors. Cells transfected with the empty pFLAG vector (HEK-FLAG/HF; white bars) served as controls. Fold change was calculated after normalization with *ACTB*. (b) Western blot was performed using specific antibodies and β-actin as endogenous control. Densitometric analysis was performed with ImageJ software (version 1.54 g; https://imagej.net/ij/). Statistical analysis was performed using Shapiro-Wilk and Student’s t-test. Error bars represent standard deviations from three independent experiments. **p < 0.01, ***p < 0.001. This figure was made using GraphPad Prism software (version 8.02; https://www.graphpad.com) and Microsoft Power Point. (Full western blot image is shown in Online Resource 1)

Overexpression of hnRNP A1 and hnRNP K was achieved upon use of the pFLAG system. HEK-293FT cells transfected with pFLAG-hnRNP A1 and pFLAG-hnRNP K were grown for 96 hours and overexpression was confirmed by RT-qPCR and western blot (Fig. 1 and Online Resource 1). RT-qPCR analysis revealed approximately 2-fold and 3-fold increases in hnRNP A1 and hnRNP K mRNA levels, respectively, compared with cells transfected with the empty pFLAG vector (Fig. 1a). At the protein level, western blot densitometric analysis indicated increases of approximately 27% for hnRNP A1 and 49% for hnRNP K (Fig. 1b).

**Fig. 2:**
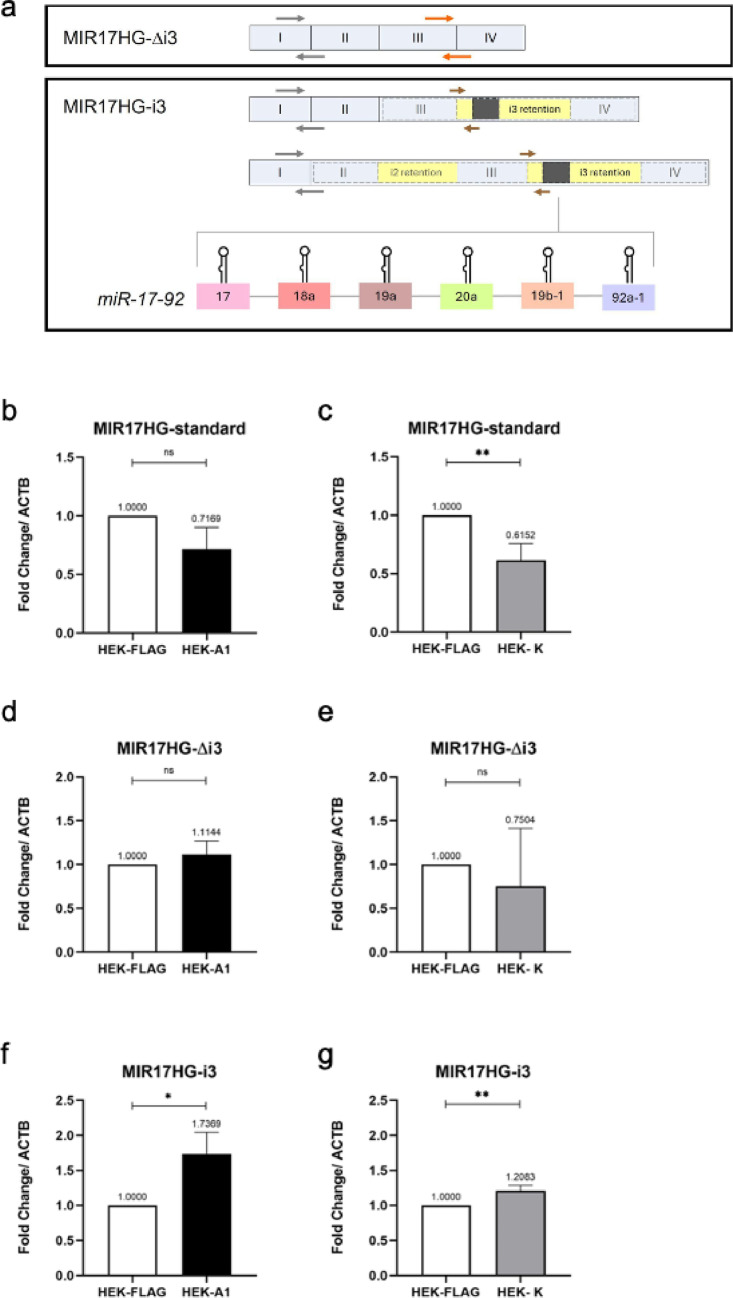
Analysis of *MIR17HG* alternative splicing. (**a**) Schematic representation of *MIR17HG* isoforms and primers used in this work. Grey rectangles represent exons. Rectangles with dashed outlines indicate the corresponding exonic regions (grey). Yellow regions represent retained intronic sequences corresponding to intron 2 (i2) and intron 3 (i3). *miR-17-92* cluster region, located within intron 3 is shown in dark grey. Arrows indicate the approximate annealing positions of the primer pairs used in this study. Grey arrows represent MIR17HG-standard primer pairs, orange arrows represent primers for MIR17HG-Δi3 isoforms and brown arrows the MIR17HG-i3 isoforms primers. The cluster miRNAs are shown in colored boxes. (**b-g**) HEK-293FT cells overexpressing hnRNP-A1 (HEK-A1; black bars) and overexpressing hnRNP K (HEK-K; grey bars) were used to assess (**b-c**) standard expression of the *MIR17HG* gene, (**d-e**) MIR17HG-Δi3 and (**f-g**) MIR17HG-i3 isoforms by RT-qPCR. Cells transfected with the empty pFLAG vector (HEK-FLAG; white bars) were used as controls. Expression values (fold change) were normalized with *ACTB*. Shapiro-Wilk and Student’s t-test were performed. Error bars represent standard deviations from three independent experiments. *p < 0.05, **p < 0.01, ns: not significant. This figure was made using GraphPad Prism software (version 8.02; https://www.graphpad.com) and Microsoft PowerPoint

To address the regulatory roles of hnRNP A1 and hnRNP K in the *MIR17HG* splicing pattern, we employed RT-qPCR to analyze MIR17HG-standard, MIR17HG-Δi3 and MIR17HG-i3 isoforms in cells overexpressing hnRNP A1 and hnRNP K. According to the last genome annotation released by Ensembl [41], *MIR17HG* has twenty different isoforms. MIR17HG-standard primers hybridize at the junction between exons 1 and 2, which is present in isoforms with and without intron 3 (i3 and Δi3), thus being important to measure *MIR17HG* basal expression levels. MIR17HG-Δi3 primers recognize exon 3 and exon 3–4 junction, amplifying isoforms lacking intron 3. MIR17HG-i3 primers hybridize to the intron 3 sequence upstream of the *miR-17-92* cluster, thus specifically detecting isoforms with intron 3 retention (Fig. 2a). Importantly, three out of four isoforms amplified with MIR17HG-i3 primers contain *miR-17-92* cluster. An overall reduction on *MIR17HG* basal expression was observed under overexpression of both proteins, although not statistically significant for hnRNP A1 (Fig. 2b-c). MIR17HG-Δi3 levels were similar among control, hnRNP A1 and hnRNP K overexpressing cells, showing a slight increase in hnRNP A1 and a mild reduction with hnRNP K, although these changes were not statistically significant (Fig. 2d-e). However, overexpression of hnRNP A1 and hnRNP K stimulated the generation of MIR17HG-i3 isoforms (Fig. 2f-g), with increases of approximately 70% and 20%, respectively. The altered levels of MIR17HG-i3 might indicate hnRNP A1 and hnRNP K modulate alternative splicing of *MIR17HG*. Another possibility is that *MIR17HG* transcripts show different stabilities in the cell, but further experiments would be necessary to explore this point. Since MIR17HG-i3 isoforms host the coding sequence for *miR-17-92* miRNAs, we reasoned overexpression of hnRNP A1 and hnRNP K would also increase the cluster miRNAs, leading to altered phenotypes.

### hnRNP A1 affects *miR-19a* processing

**Fig. 3:**
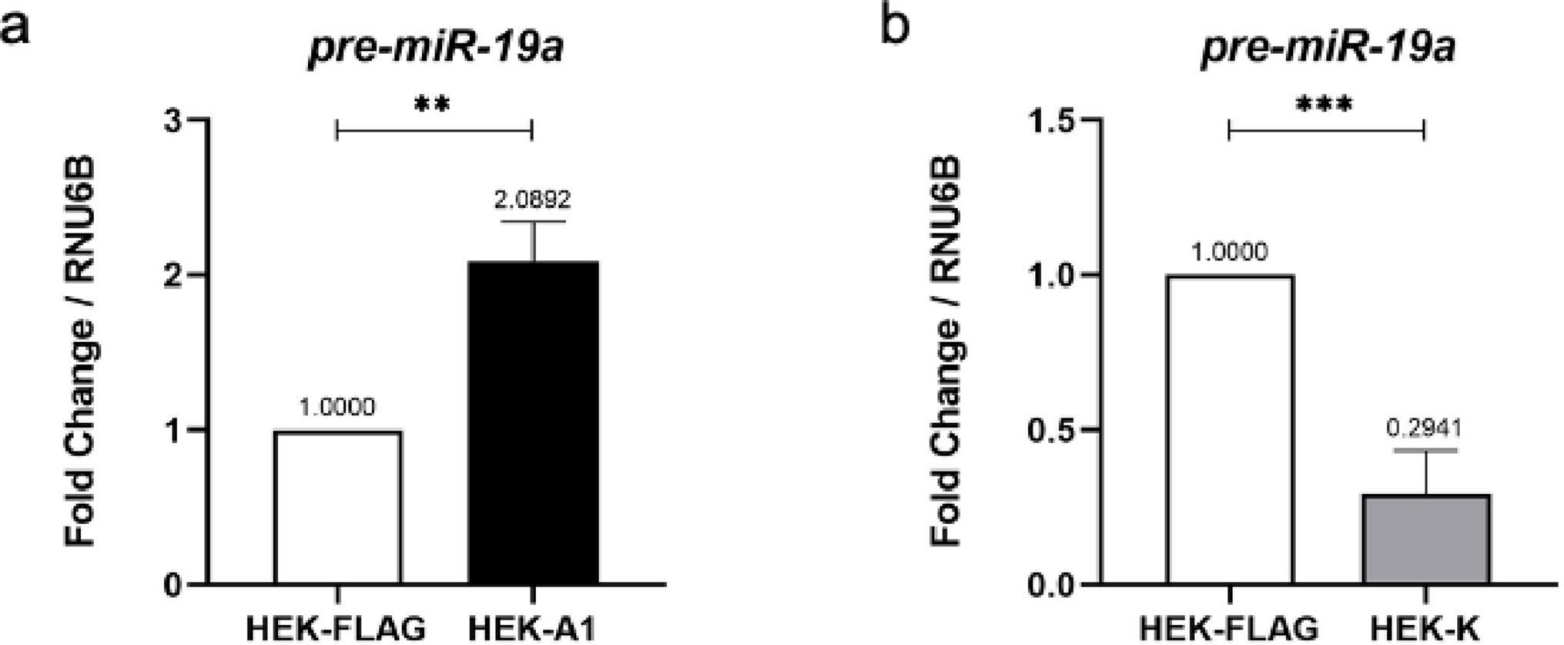
Quantification of *pre-miR-19a* levels by RT-qPCR in cells overexpressing (**a**) hnRNP A1 and (**b**) hnRNP K. HEK-293FT cells overexpressing hnRNP A1 are represented by black bars (HEK-A1), and those overexpressing hnRNP K by grey bars (HEK-K). White bars represent control cells transfected with the empty pFLAG vector (HEK-FLAG). Expression values (fold change) were normalized with the expression of *RNU6B*. Error bars represent standard deviations from three independent experiments. **p < 0.01; ***p < 0.001. This figure was made using GraphPad Prism software (version 8.02; https://www.graphpad.com)

MIR17HG-i3 isoforms contain *miR-17-92* miRNAs cluster. Thus, we reasoned that increased expression of these isoforms would also lead to elevated expression of the individual *miR-17-92* miRNAs. To address that, we investigated the expression of *miR-19a*, one of the most oncogenic miRNAs of this cluster [27]. The precursor of *miR-19a* was assessed by RT-qPCR using specific primers (Table 1). Upon hnRNP A1 overexpression, *pre-miR-19a* expression increased by 2-fold (Fig. 3a). A similar result was previously observed in papillary thyroid carcinoma cells (BCPAP cell line) [15]. Altogether, these results confirm the active participation of hnRNP A1 in *MIR17HG* splicing and suggest that this protein might also affect the generation of *miR-19a*.

However, overexpression of hnRNP K inhibits *pre-miR-19a* generation, despite the increase on MIR17HG-i3 isoforms (Fig. 3b). *In silico* analysis revealed hnRNP K binding sites distributed along intron 3, including one site in proximity to the 5’ end of the miRNA cluster and another within a miRNA coding sequence, suggesting that this protein may bind to pre-miRNA sequence during RNA processing (Online Resource 2). It is possible that hnRNP K binding during splicing prevents or slows *pre-miR-19a* generation, but further experiments are required to confirm this hypothesis.

**Fig. 4:**
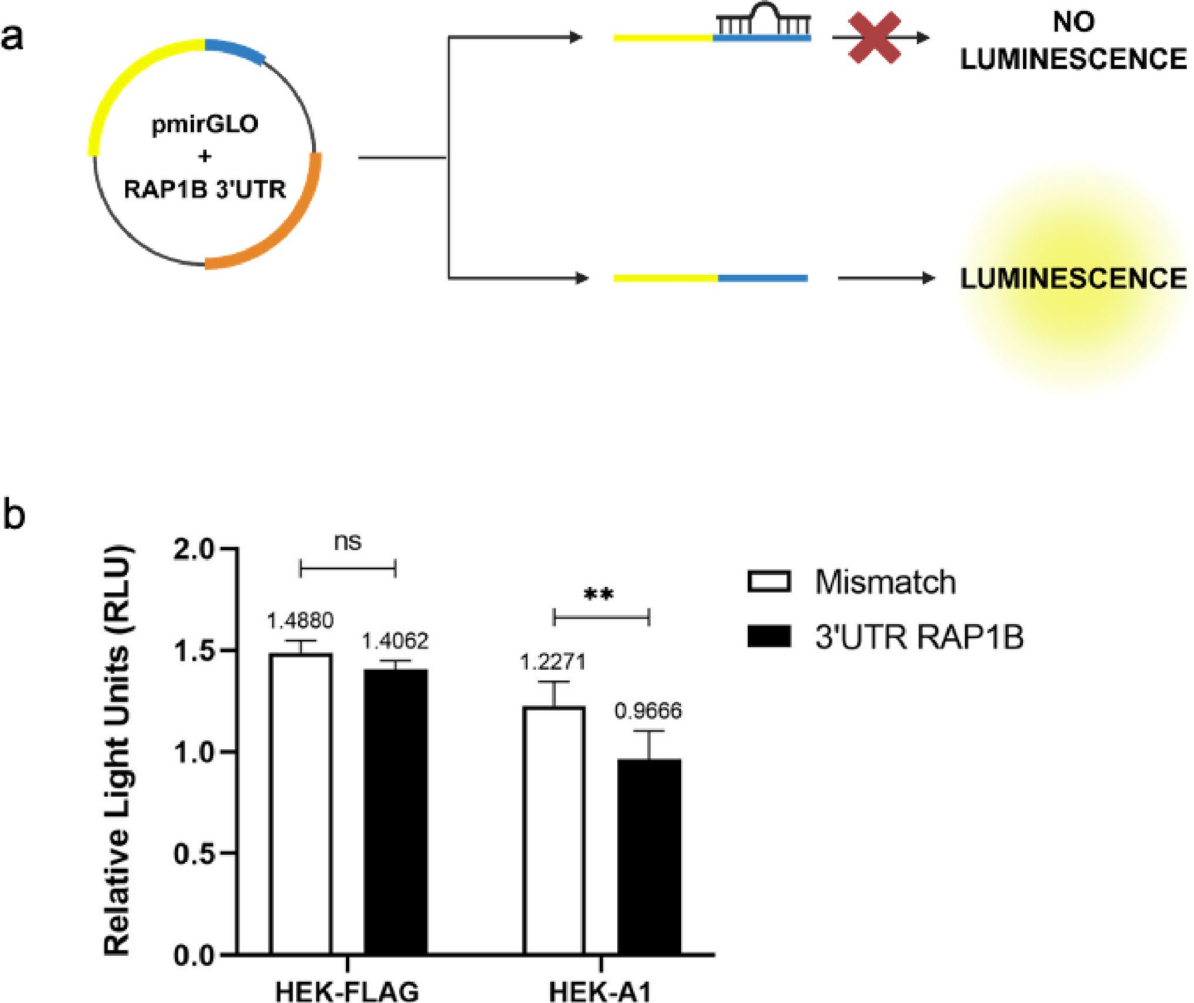
Increased *miR-19a* recognition of RAP1B 3’UTR in cells overexpressing hnRNP A1. (**a**) Schematic representation of the luciferase reporter assay using pmirGLO-RAP1B plasmid. Recognition of the miRNA at its binding site within the inserted 3′ UTR sequence (blue) leads to reduced firefly luciferase expression (coding sequence marked in yellow). (**b)** Relative luciferase activity from pmirGLO reporter vectors containing either the RAP1B 3′ UTR (3’UTR-RAP1B; black bars) or a scrambled control sequence (Mismatch; white bars) in HEK-293FT cells co-transfected with either pFLAG-hnRNP A1 or the empty pFLAG vector. Firefly luciferase activity was normalized to Renilla luciferase (FL/RL) and expressed as relative light units (RLU), using empty pmirGLO as internal control. Statistical analysis was performed using two-way ANOVA followed by Turkey’s multiple comparisons post-test. Data represents the mean ± SD of five independent biological replicates. **p < 0.01. This image was created using GraphPad Prism software (version 8.02; https://www.graphpad.com) and BioRender (Coltri, P. (2026) https://BioRender.com/eyuvdnh)

To address whether *miR-19a* was correctly processed after increased expression promoted by hnRNP A1 overexpression, we used a reporter system based on luciferase expression (Fig. 4a). A plasmid containing part of RAP1B 3’UTR fused to luciferase was used [29]. Our results revealed significantly lower luciferase activity for RAP1B than for the mismatch group in cells overexpressing hnRNP A1 (Fig. 4b). No significant difference between the two target sequences was detected in the control group transfected with empty pFLAG vectors. These results confirm an enhanced repression of the RAP1B target on cells overexpressing hnRNP A1. We interpret this effect as evidence that the increased generation of *pre-miR-19a*, observed by RT-qPCR, is followed by increased levels of functional *miR-19a* (Fig. 4b). Altogether, our results indicate that *MIR17HG* splicing regulation performed by hnRNP A1 increases MIR17HG-i3 isoforms generation, which correlates with higher levels of functional *miR-19a*.

### Overexpression of hnRNP A1 and hnRNP K positively impacts cell proliferation

**Fig. 5:**
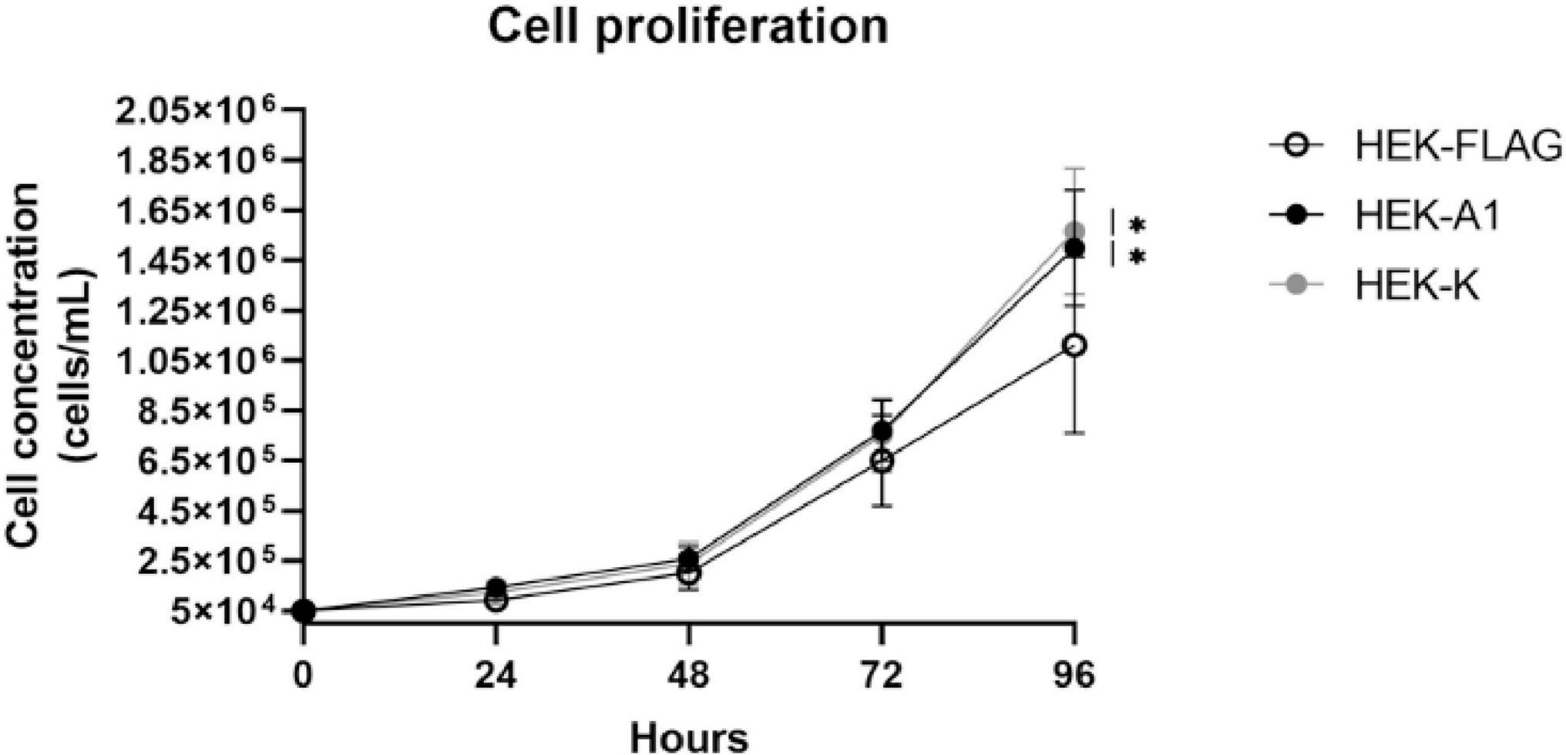
Cell proliferation assay of HEK-293FT cells. HEK-293FT cells transfected with pFLAG-hnRNP A1 (black circles) and pFLAG-hnRNP K (grey circles) vectors are shown. HEK-293FT cells transfected with the empty pFLAG vector (white circles) were used as controls. Cells were counted at 24, 48, 72, and 96 hours after plating using 0.4% Trypan Blue solution. Values represent the mean of technical replicates from four biological replicates per group. Error bars represent standard deviations from each experiment. Shapiro-Wilk and Student’s t-test were performed. *p < 0.05. This image was created using GraphPad Prism software (version 8.02; https://www.graphpad.com)

As we have previously observed in BCPAP cells [15], overexpression of hnRNP A1 in HEK-293FT cells led to increased cell proliferation (Fig. 5). This is also consistent with the stimulation of pro-oncogenic features. HEK-293FT cells overexpressing hnRNP A1 and hnRNP K showed 35% and 40% increase in cell number after 96 hours, statistically significant increases in comparison to those observed in the FLAG control. Cell death rates were approximately 12.1% for the hnRNP A1 group, 5.18% for the hnRNP K group, and 6.3% for the negative control group, with no statistically significant differences among them, indicating that most cells remained viable throughout the experiment. As for hnRNP K, the result suggests that it promotes cell growth, similarly to hnRNP A1. We interpret that hnRNP K is possibly affecting cell proliferation through other pathways related to cell cycle control, but not necessarily through *miR-17-92* cluster. Indeed, hnRNP K knockdown affected the transcriptional regulation of cyclin D1, resulting in reduction of proliferation in bladder cancer cells [21]. Consistently, hnRNP K overexpression has been related to control cell proliferation in HeLa [42] and lung cancer [19].

## Discussion

Pre-mRNA splicing is an essential step in the control of gene expression, defining cellular responses, signaling pathways and affecting phenotypes. We previously reported elevated levels of *miR-17-92* miRNAs in papillary thyroid cancer cells upon hnRNP A1 overexpression, especially *miR-17a* and *miR-18a* [15]. This work aimed to investigate the potential regulatory axis involving alternative splicing regulation of *MIR17HG*, hnRNPs and *miR-19a*, considered one of the most oncogenic miRNAs in this cluster [27]. We investigated whether overexpression of hnRNP A1 and hnRNP K could impact the splicing of the *miR-17-92* host gene *MIR17HG*, potentially affecting the isoform expression pattern and, therefore, the biogenesis of miRNAs from this cluster. Our results contribute to understanding the roles of hnRNPs A1 and K in the alternative splicing of *MIR17HG* and their impact on *miR-19a* biogenesis, as we discuss below.

Overexpression of both hnRNP A1 and hnRNP K was associated with a mild reduction in MIR17HG-standard levels, whereas MIR17HG-i3 expression increased. Because MIR17HG-standard reflects overall transcript abundance independently of isoform composition, these findings suggest that the increased abundance of MIR17HG-i3 is unlikely to result from a general increase in *MIR17HG* transcription. Instead, they are consistent with a shift in *MIR17HG* transcript processing. Given the established roles of hnRNP proteins in splice-site selection [8, 11], hnRNP A1 and hnRNP K may promote the generation of MIR17HG-i3 through modulation of alternative splicing. Another possibility is that the increased levels of MIR17HG-i3 reflect enhanced transcript stability rather than, or in addition to, altered splicing decisions. Together, our findings indicate that hnRNP A1 and hnRNP K influence *MIR17HG* isoform expression pattern, favoring MIR17HG-i3.

MIR17HG-i3 isoforms retain the intronic region containing the *miR-17-92* cluster, which encodes six independently processed miRNAs [15, 27, 28] with distinct mRNAs targets, including RAP1B, MCL1, BCL1L11 (BIM), E2F1, and TGFβR2, thereby affecting cell proliferation, death and migration, ultimately impacting tumoral characteristics [32, 37, 43, 44]. Although processing of intronic miRNAs could occur from both intron-retained transcripts and excised intron lariats [29, 45–48], there is no direct evidence that *miR-17-92* biogenesis occurs through intron-lariat processing [29, 49]. Our data shows that MIR17HG-Δi3 expression remained comparable across all conditions. Therefore, the contribution of lariat-derived processing would not be expected to differ substantially between groups. Together with the observed increase in MIR17HG-i3 levels, these findings support the interpretation that downstream changes in miRNA expression could be associated with the elevated levels of these isoforms. Accordingly, we expected enhanced pre-miRNA generation and, consequently, more mature miRNAs levels. To determine whether the effects of hnRNP A1 and hnRNP K on *MIR17HG* splicing were associated with downstream changes in miRNA biogenesis, we selected *miR-19a* for further analysis, given its prominent role in mediating the oncogenic functions of the *miR-17-92* cluster [27].

In cells overexpressing hnRNP A1, increased expression of the MIR17HG-i3 isoforms resulted in higher levels of *pre-miR-19a*, indicating that this protein facilitates processing of this pre-miRNA. This is consistent with previous results from our group and others, which reported increased levels of *miR-17-92* miRNAs upon hnRNP A1 overexpression [15, 18]. *miR-19a* levels were addressed by luciferase reporter assays using RAP1B as the target sequence. Reduced luciferase activity with RAP1B target was observed in cells overexpressing hnRNP A1, indicating a specific effect, and confirming increased synthesis of functional *miR-19a*. Increased levels of *miR-19a* have been reported in different tumor types, including cervical, pancreatic, lung and colorectal cancers [30, 32, 50, 51], indicating that this miRNA might contribute to pro-tumorigenic characteristics [30, 52].

Overexpression of hnRNP K led to increased levels of MIR17HG-i3 isoforms, as observed with hnRNP A1 and, accordingly, *pre-miR-19a* levels were expected to mirror the same expression patterns. Interestingly, reduced levels of *pre-miR-19a* were observed, indicating its processing is diminished under this condition. It is possible that binding sites for hnRNP K within intron 3 (Online Resource 2) could interfere with *pre-miR-19a* biogenesis by, for example, changing the structural organization of the pri-miRNA, potentially limiting Drosha/DGCR8 access to this intronic region and consequently impairing microprocessor-mediated processing [53, 54], but further specific experiments are required to confirm that.

Interestingly, although hnRNP A1 and hnRNP K displayed opposite effects on *pre-miR-19a* expression, both proteins promoted increased cell proliferation. In HEK-293FT cells, the proliferative phenotype observed upon hnRNP A1 overexpression suggests that important cell cycle regulators were stimulated, consistent with the correlation of this RBP with tumoral progression [20–22, 30]. Likewise, hnRNP K overexpression resulted in increased cell proliferation, in agreement with studies reporting its correlation with colorectal and gastric tumors progression and malignancy [20–22]. Furthermore, hnRNP K has been implicated in the transcriptional regulation of the proto-oncogene *MYC* [55], highlighting its ability to influence tumorigenic processes through multiple molecular pathways. Therefore, the proliferative effects observed following overexpression of these hnRNPs may involve regulatory mechanisms beyond the modulation of *MIR17HG* splicing and *miR-19a* biogenesis.

## Conclusion

Our results indicate that both hnRNP A1 and hnRNP K impact the *MIR17HG* splicing pattern, favoring isoforms that retain the intron containing the *miR-17-92* cluster. Interestingly, the downstream consequences of this splicing shift on miRNA biogenesis differ between the two RBPs. While hnRNP A1 correlates with increased levels of *miR-19a*, hnRNP K exerts the opposite effect on this miRNA. Future experiments comparing the microprocessor and spliceosome activities will be important to further dissect the interplay of these macromolecular complexes.

## Supplementary Information

Below is the link to the electronic supplementary material.

Supplementary Material 1

## Data Availability

The data that support the findings of this study are available from the corresponding author upon reasonable request.

## Abbreviations

FL: Firefly luciferase
hnRNP: Heterogeneous ribonucleoprotein
miRNA: microRNA
RBP: RNA binding protein
RL: Renilla luciferase
RLU: Relative light units
RRM: RNA recognition motif
RGG box: Arginine-glycine-rich domain
snRNA: Small nuclear RNA
