# Supplementary material for: hnRNP A1 controls MIR17HG alternative splicing and maturation of miR-19a
Source: Mol Biol Rep. 2026 Aug 12;53(1):1386. doi: 10.1007/s11033-026-12580-6 (PMC13468856; doi:10.1007/s11033-026-12580-6)
Supplement: Supplementary file 1 [file 11033_2026_12580_MOESM1_ESM.pdf]

hnRNP A1 controls *MIR17HG* alternative splicing and maturation of *miR-19a*

Vivian Maltese Munhoz[1], Letícia Oliveira Rojas Cruz[1], Patricia Pereira Coltri[1]*

[1]Departamento de Biologia Celular e do Desenvolvimento, Instituto de Ciências Biomédicas, Universidade de São Paulo, São Paulo, SP, Brazil, 05508-000, +55-11-30910980

*Corresponding author: P. Coltri, coltri@usp.br

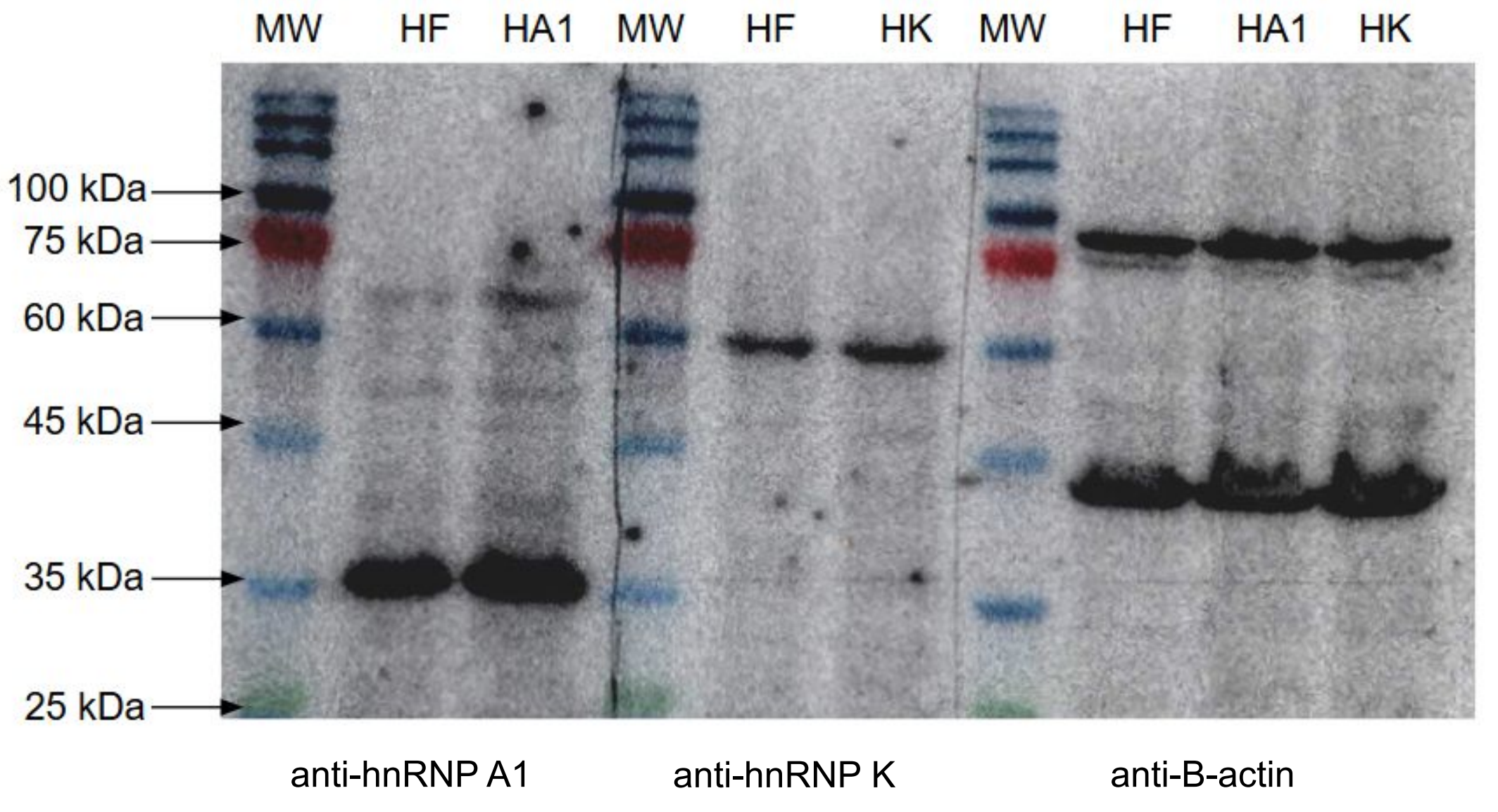

Online Resource 1: Representative full raw western blot image performed with HEK-293FT transfected with pFLAG (HEK-FLAG), pFLAG-hnRNP A1 (HEK-A1) and pFLAG-hnRNP K (HEK-K), using anti-hnRNP A1 (Proteintech), anti-hnRNP K (Proteintech) and anti-β-actin (Sigma). MW. Molecular weight pre-stained marker (Thermo)

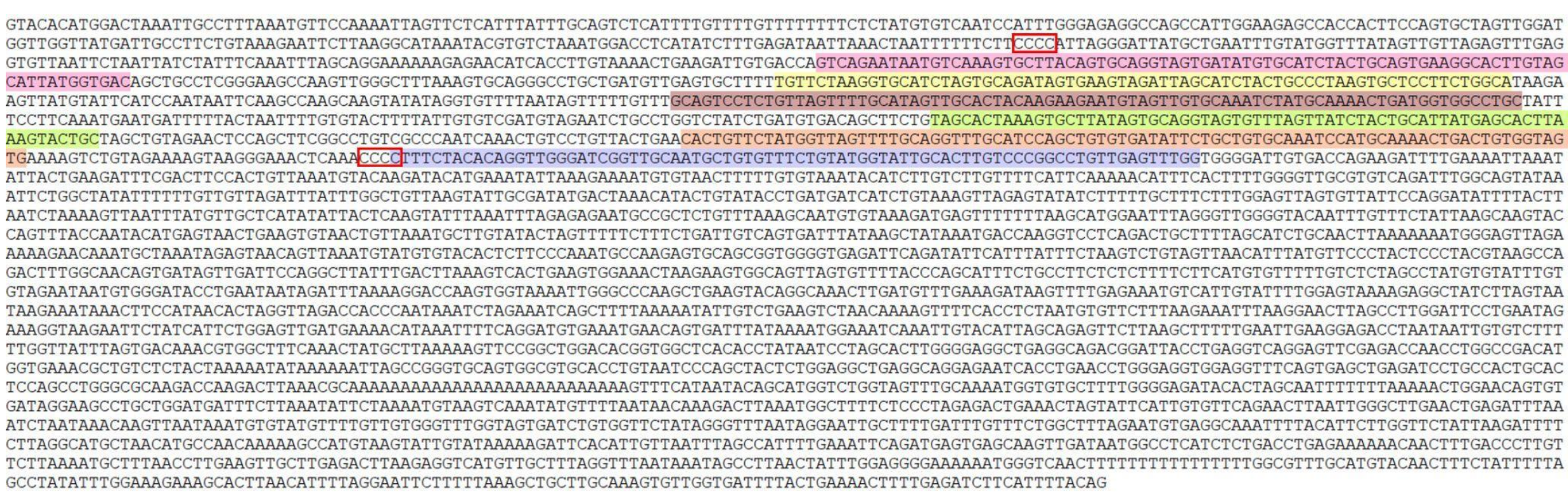

Online Resource 2: *In silico* analysis of hnRNP K binding sites within the intron 3 sequence in MIR17HG-i3 isoforms. The miRNAs of the *miR-17-92* cluster are highlighted in different colors: the *pre-miR-17* sequence is shown in pink; the *pre-miR-18a* sequence in yellow; the *pre-miR-19a* sequence in brown; the *pre-miR-20a* sequence in green; the *pre-miR-19b*-1 sequence in orange; and the *pre-miR-92a-1* sequence in purple. The red rectangles highlight the poly-C regions, to which the hnRNP K protein shows a preferential interaction [7]. GeneCards and NCBI databases were used as references for the *in silico* analysis.
